# Design of *Lactococcus lactis* Strains Producing Garvicin A and/or Garvicin Q, Either Alone or Together with Nisin A or Nisin Z and High Antimicrobial Activity against *Lactococcus garvieae*

**DOI:** 10.3390/foods12051063

**Published:** 2023-03-02

**Authors:** Javier Feito, Carlos Araújo, Sara Arbulu, Diogo Contente, Beatriz Gómez-Sala, Lara Díaz-Formoso, Estefanía Muñoz-Atienza, Juan Borrero, Luis M. Cintas, Pablo E. Hernández

**Affiliations:** 1 Grupo de Seguridad y Calidad de los Alimentos por Bacterias Lácticas, Bacteriocinas y Probióticos (Grupo SEGABALBP), Sección Departamental de Nutrición y Ciencia de los Alimentos (Higiene y Seguridad Alimentaria), Facultad de Veterinaria, Universidad Complutense de Madrid, Avda. Puerta de Hierro, s/n., 28040 Madrid, Spain; 2 APC Microbiome Ireland, University College Cork, T12 K8AF Cork, Ireland; 3 Teagasc Food Research Centre, Moorepark, P61 C996 Cork, Ireland

**Keywords:** bacteriocins, garvicin, nisin, *Lactococcus garvieae*, lactic acid bacteria (LAB), heterologous production, *Lactococcus lactis*, probiotics, paraprobiotics, postbiotics

## Abstract

*Lactococcus garvieae* is a main ichthyopathogen in rainbow trout (*Oncorhynchus mykiss*, Walbaum) farming, although bacteriocinogenic *L. garvieae* with antimicrobial activity against virulent strains of this species have also been identified. Some of the bacteriocins characterized, such as garvicin A (GarA) and garvicin Q (GarQ), may show potential for the control of the virulent *L. garvieae* in food, feed and other biotechnological applications. In this study, we report on the design of *Lactococcus lactis* strains that produce the bacteriocins GarA and/or GarQ, either alone or together with nisin A (NisA) or nisin Z (NisZ). Synthetic genes encoding the signal peptide of the lactococcal protein Usp45 (SP*_usp45_*), fused to mature GarA (*lgnA*) and/or mature GarQ (*garQ*) and their associated immunity genes (*lgnI* and *garI*, respectively), were cloned into the protein expression vectors pMG36c, which contains the P*_32_* constitutive promoter, and pNZ8048c, which contains the inducible P*_nisA_* promoter. The transformation of recombinant vectors into lactococcal cells allowed for the production of GarA and/or GarQ by *L. lactis* subsp*. cremoris* NZ9000 and their co-production with NisA by *Lactococcus lactis* subsp. *lactis* DPC5598 and *L. lactis* subsp. *lactis* BB24. The strains *L. lactis* subsp*. cremoris* WA2-67 (pJFQI), a producer of GarQ and NisZ, and *L. lactis* subsp*. cremoris* WA2-67 (pJFQIAI), a producer of GarA, GarQ and NisZ, demonstrated the highest antimicrobial activity (5.1- to 10.7-fold and 17.3- to 68.2-fold, respectively) against virulent *L. garvieae* strains.

## 1. Introduction

Bacteriocins produced by lactic acid bacteria (LAB) have been largely valued as potential food preservatives, and the LAB producers of bacteriocins (bacteriocinogenic strains) have been valued as potential starter, protective, probiotic, paraprobiotic and postbiotic cultures [1,2,3]. Moreover, concerns regarding the increase in antimicrobial resistances (AMRs) confer bacteriocins and the bacteriocinogenic LAB unlimited possibilities for applications in the food industry, human and veterinary medicine and the animal production field [4,5,6]. Microbial-derived biotics, including bacteriocins, are recognized as functional components of natural and bioengineered probiotic, paraprobiotic and postbiotic cultures [2,7,8]. Bacteriocins, including nisin A (NisA) and nisin Z (NisZ), drive the apoptosis of cancer cells and show low toxicity toward normal cells, making them promising anticancer candidates to replace or be combined with conventional therapeutic agents [9,10,11]. Bacteriocinogenic LAB and the bacteriocins they produce may also have an impact in the modulation of the microbiota and immune system of their host [12,13].

Most LAB bacteriocins are synthesized as biologically inactive precursors that contain an N-terminal extension, whereas the mature peptides are often cationic, amphiphilic, membrane-permeabilizing molecules. The N-terminal extensions of most bacteriocins are of the so-called double-glycine type (leader sequence) and are cleaved off concomitantly with export across the cytoplasmic membrane by dedicated, ATP-binding cassette transporters (ABC-transporters) and their accessory proteins [14]. However, a few bacteriocins contain N-terminal extensions of the Sec-type (signal peptide), which are proteolytically cleaved concomitantly with peptide externalization by the general secretory pathway (GSP) or the Sec-dependent pathway [15]. Several bacteriocins have been shown to be synthesized without N-terminal sequences and represent bacteriocins with a novel secretion mechanism [16].

The mature bacteriocins are often classified into three main classes: class I, or lantibiotics, which have post-translationally modified amino acid residues, class II bacteriocins, which have unmodified amino acid residues, and class III large, heat-labile bacteriocins [17,18]. However, the class I bacteriocins are currently included in the group of ribosomally synthesized and post-translationally modified peptides (RiPPS) [18,19,20]. The class II bacteriocins can be further divided into the subclasses pediocin-like (class IIa), two-peptide (class IIb), leaderless (class IIc) and the non-pediocin single (class IId) bacteriocins [18,21]. In most cases, bacteriocin production appears to be regulated and involves a quorum-sensing mode of regulation [22,23]. The production of nisin is autoregulated by NisK, a sensor kinase protein, and NisR, a response regulator, which regulate transcription via signal transduction by a two-component regulatory system. The induction of NisA or NisZ is likely to be dependent on their interaction with NisK [23]. The NisI and the ATP-binding cassette transporter NisEFG support the immunity of the producer cells to nisin [24].

The presence of LAB in many ecological niches, including fish and their rearing environment, is well characterized, and the antimicrobial activity and the probiotic potential of the isolated strains is emphasized [25,26,27,28,29]. However, despite the beneficial role of most LAB, the species *Lactococcus garvieae* has been identified as the aetiological agent of lactococcosis, a major, re-emerging and widely distributed ichthyopathology that causes a hyperacute haemorrhagic septicaemia and relevant economic losses in rainbow trout (*Oncorhynchus mykiss*, Walbaum) farming [25,30]. The reported human cases of pathologies associated with this bacterium are increasing, and its zoonotic potential has been accepted [31,32,33].

Bacteriocinogenic *L. garvieae* that demonstrate an antagonistic activity against virulent *L. garvieae* strains have been isolated. Their bacteriocins have been characterized, such as garvicin A [34], garvicin AG1 and garvicin AG2 [35], garvicin KS [36], garvicin L1-5 [37], garvicin ML [38] and garvieacin Q [39]. GarA, produced by *L. garvieae* 21881, is encoded in the plasmid pGL5, which holds the structural gene of GarA (*lgnA*), its putative immunity protein (*lgnI*) and the ABC-transporter and its accessory proteins (*lgnC* and *lgnD*). GarA is synthesized as a 63 amino acid precursor with a typical double-glycine leader peptide. Once processed, this peptide results in a 43-aa mature peptide with a theoretical molecular mass of 4645.5 Da. GarA has a narrow activity spectrum and is limited only to other *L. garvieae* strains, suggesting that its mechanism of action is based on the inhibition of cell division, most likely by inhibiting septum formation in target cells [34]. GarQ is produced by *L. garvieae* BCC 43588 and is encoded as the GarQ structural gene (*garQ*), its putative immunity protein (*garI*) and the ABC-transporter (*garT*). GarQ shows a wide spectrum of antimicrobial activity against LAB and other potentially foodborne pathogenic strains [39]. GarQ is synthesized as a 70-aa precursor with a double-glycine cleavage site, resulting in mature GarQ of 50-aa and a theoretical molecular mass of 5340 Da. GarQ utilizes the IID and IIC subunits of the mannose phosphotransferase system (Man-PTS) as a receptor. It kills target bacteria by disrupting the membrane integrity, mainly locking the Man-PTS into a conformation that leads to the formation of a constitutively open pore [40,41]. NisZ, a 34 amino acid pentacyclic peptide naturally produced by *L. lactis*, exhibits antimicrobial activity against several Gram-positive and Gram-negative bacteria [42], and the bacteriocin exerts its antimicrobial activity by both pore formation and the inhibition of cell wall synthesis through specific binding to lipid II, which is an essential precursor of the bacterial cell wall [43].

The cloning and heterologous expression of bacteriocins by LAB, particularly *Lactococcus lactis*, has proven to be a promising approach for obtaining microbial cell factories with a potent antimicrobial activity [44,45,46,47,48]. Moreover, the simultaneous production of bacteriocins of different classes and/or subclasses and distinct modes of action may not only improve their antimicrobial activity and spectrum in a synergistic fashion but may also reduce the presence of bacteria that are resistant to their antagonistic activity [49,50,51]. In this study, we have proceeded to the design and expression in different *L. lactis* strains of up to three different bacteriocins with antimicrobial activity against virulent *L. garvieae*. These bacteriocins, namely, GarA, GarQ and NisA/NisZ, show different modes of action and other well-described beneficial effects, such as the anticarcinogenic effect of NisA/NisZ and its ability to modulate the microbiota and regulate the immune system of its host. Thus, synthetic genes that encode the signal peptide of the lactococcal secreted protein Usp45 (SP*_usp45_*), fused to either mature GarA (*lgnA*) with its putative immunity gene (*lgnI*) and/or to mature GarQ (*garQ*) with its immunity gene (*garI*), were cloned into the protein expression vectors pMG36c which encodes the P*_32_* constitutive promoter and in plasmid pNZ8048c, under control of the inducible P*_nisA_* promoter. Recombinant *L. lactis* strains were then obtained, and their antimicrobial activity against virulent *L. garvieae* was determined.

## 2. Materials and Methods

### 2.1. Bacterial Strains, Plasmids and Growth Conditions

The bacterial strains and plasmids used in this study are listed in Table 1. The *L. lactis* strains were grown at 30 °C in M17 broth (Oxoid Ltd., Basingstoke, UK) supplemented with 0.5% (*w*/*v*) glucose (GM17). *Pediococcus damnosus* CECT4797 was grown in MRS broth (Oxoid Ltd.) at 30 °C. *Escherichia coli* JM109 (Promega, Madison, WI, USA) was grown in Luria–Bertani (LB) broth (Oxoid Ltd.) at 30 °C with shaking. Chloramphenicol (Sigma-Aldrich, St. Louis, MO, USA) was added at 20 µg/mL to select growth of *E. coli* and at 5 µg/mL for the selection of the recombinant lactococcal strains. The cell dry weights of the late exponential phase cultures were determined gravimetrically. Agar plates were made by the addition of 1.5% (*w*/*v*) agar (Oxoid) to the liquid media.

### 2.2. Basic Genetic Techniques and Enzymes

Synthetic gene fragments were designed from the described amino acid sequence of the bacteriocin GarA (*lgnA*) and GarQ (*garQ*), as well as those from their putative immunity proteins GarAI (l*gnI*) and GarQI (*garI*), respectively. In addition, the leader peptide of the native bacteriocins was replaced by the signal peptide of the secreted protein Usp45 (SP*_usp45_*), a Sec-dependent protein produced by *L. lactis* MG1363 [45,57]. Similarly, additional sequences containing the *Sac*I cleavage site and the P*_32_* ribosome binding site (RBS) as well as the *Sac*I/*Hind*III or the *Bsp*HI/*Hind*III enzyme restriction cleavage sites were added at the 5′ and 3′ ends, respectively, of the designed synthetic gene fragments. Their codon usage was adapted for its expression by *L. lactis*. GeneArt^®^ supplied the synthetic genes into the carrier plasmid pMA-T (Life Technologies S.A., Madrid, Spain). The protein expression vectors pMG36c and pNZ8048c were purified from *E. coli* JM109 by using the NucleoSpin Plasmid Kit (Macherey-Nagel, Düren, Germany). DNA restriction enzymes were supplied by New England Biolabs (Beverly, MA, USA). Ligations were performed with the T4 DNA ligase (Invitrogen, Walthman, MA, USA). Electrocompetent *L. lactis* subsp. *cremoris* NZ9000, *L. lactis* subsp*. cremoris* WA2-67, *L. lactis* subsp. *lactis* DPC5598 and *L. lactis* subsp. *lactis* BB24 cells were obtained after successive growth in the SGGM17 medium, which consisted of of M17 (Oxoid Ltd.) supplemented with 0.5 M sucrose, glucose (0.5%; *w*/*v*) and glycine (2%; *w*/*v*). The cultures were centrifuged and resuspended in a cold wash buffer containing glycerol (20%; *v*/*v*) and 0.5 M sucrose. Aliquots of 50 µL were stored at −80 °C until further use.

### 2.3. Recombinant Plasmids Derived from pMG36c and Transformation into L. lactis Hosts

The primers and PCR products used for the construction of the pMG36c-derived vectors are listed in Table S1. PCR product amplifications were performed in 50 μL reaction mixtures that contained 20 ng of the synthetic gene fragments included in the carrier pMA-T vectors, 70 pmol of each primer, 1 U of Velocity DNA polymerase (Bioline Reagents, Ltd., London, UK), 10 µL of Hi-Fi buffer and 1.5 µL of 30 mM dNTP’s mix in a MJ Mini Gradient Thermal Cycler (BioRad Laboratories). PCR cycling conditions were as follows: denaturation at 98 °C (2 min), 35 cycles of denaturation–annealing–extension (98 °C for 30 s, 60 °C for 30 s and 72 °C for 30 s, respectively) and a final extension step at 72 °C (5 min). The PCR-generated fragments were purified using a NucleoSpin^®^ Gel and PCR clean-up kit (Macherey-Nagel) for cloning and nucleotide sequencing. When required, PCR amplifications were sequenced using the ABI PRISM^®^ BigDye^®^ Terminator cycle sequence reaction kit and the automatic DNA sequencer ABI PRISM, model 377 (Applied Biosystems, Foster City, CA, USA) at the Unidad de Genómica (CAI Técnicas Biológicas, UCM, Madrid, Spain). Digestion of the amplified PCR products with *SacI*/*Hind*III permitted the ligation of the resulting restriction fragments into pMG36c, which was digested with the same enzymes. The resulting pMG36c-derived vectors were transformed into competent lactococcal hosts and electrotransformed with a Gene Pulser^TM^ and Pulse Controller apparatus (Bio-Rad Laboratories, Hercules, CA, USA), according to a previously described procedure [58]. Transformed cells containing the pMG36c-derived vectors pJFAI, pJFQI, pJFAIQI and pJFQIAI (Table 1), were selected for their growth with chloramphenicol and evaluated for their bacteriocinogenity. The total bacterial DNA from the transformed lactococcal strains was purified using the InstaGene Matrix (BioRad Laboratories). It was then submitted to PCR using the primers MGPJ-F and MGPJ-R. The sequencing of the generated PCR products was performed at the Unidad de Genómica (CAI Técnicas Biológicas, UCM).

### 2.4. Recombinant Plasmids Derived from pNZ8048c and Transformation into L. lactis Hosts

The primers and PCR products used for the construction of the pNZ8048c-derived vectors are listed in Table S1. The initial PCR amplification of the designed gene fragments into carrier pMA-T vectors was performed with primers GARF-BSPHI and GARAIM-R, which were designed to provide the restriction cleavage site for *Bsp*HI/*Hind*III. The digestion of the amplified PCR products with *Bsp*HI/*Hind*III permitted the ligation of the resulting restriction fragments into pNZ8048c, which was digested with *Nco*I and *Hind*III. However, it should be highlighted that construction of the pNZ8048c-derived vectors carrying GarQ or GarQ and GarA and their respective immunity proteins was achieved using a novel, PCR-based, restriction-enzyme-free cloning, or ABC cloning, method [59]. Briefly, the procedure implies the PCR amplification of three overlapping fragments, two from the pNZ8048c vector and one from the previously designed synthetic gene fragments, to generate a single, circular, pNZ8048c-derived vector by using a pair of overlapping primers. For the amplification of the appropriate gene fragments, 50 µL PCR reactions containing 100 ng of plasmid pNZ8048c or the synthetic gene fragments included in the carrier pMA-T vectors, 0.5 µmol of each primer and 25 µL of Phusion Hot Start II High-Fidelity PCR Master Mix (Thermo Scientific, Waltham, MA, USA) were used. PCR cycling conditions were as follows: one initial denaturation step at 98 °C (30 s), 30 cycles of denaturation–annealing–extension (98 °C for 10 s, 49.1–65.8 °C for 20 s and 72 °C for 25 s, respectively) and a final extension step at 72 °C for 5–10 min). Overlapping PCRs were carried out using the three corresponding fragments as templates. Specifically, 1.5 × 10^10^ copies of fragments derived from amplification of pNZ8048c and 3 × 10^10^ copies of fragments obtained from amplification of the designed synthetic genes were used. Agarose gel electrophoresis, visualization and sequencing of the generated PCR products were performed essentially as described for the construction of the pMG36c-derived vectors. The resulting pNZ8048c-derived vectors were transformed into competent lactococcal hosts, and the transformed cells containing the pNZ8048c-derived vectors pNJFAI, pNJQI and pNJFQIAI (Table 1) were selected for their growth with chloramphenicol and evaluated for their bacteriocinogenicity. Bacterial DNA from the lactococcal transformed cells was submitted to PCR amplification with the primers NZPJ-F and NZPJ-R. The sequencing of the generated PCR products was performed at the Unidad de Genómica (CAI Técnicas Biológicas, UCM).

### 2.5. Antimicrobial Activity of the Recombinant L. lactis Strains

The direct antimicrobial activity of colonies from the recombinant lactococcal strains was examined by a stab-on-agar test (SOAT) as previously described [60]. When appropriate, cultures were induced with nisin A (Sigma-Aldrich) at a final concentration of 10 ng/mL for the production of the cloned bacteriocins. Cell-free culture supernatants (CFS) were obtained by the centrifugation of cultures at 12,000× *g* at 4 °C for 10 min, adjusted to pH 6.2 with 1 M NaOH, filtered through 0.22 μm pore-size syringe filters (Sartorius, Göttingen, Germany) and stored at −20 °C until further use. The antimicrobial activity of the supernatants was determined by an agar diffusion test (ADT). It was further quantified by a microtiter plate assay (MPA) as previously described [60]. For the MPA, the growth inhibition of sensitive cultures was measured spectrophotometrically at 620 nm with a FLUOstar OPTIMA (BMGLabtech, Ortenberg, Germany) plate reader. One bacteriocin unit (BU) was defined as the reciprocal of the highest dilution of the bacteriocin that caused a growth inhibition of 50% (50% of the turbidity of the control culture without bacteriocin).

### 2.6. Purification of Bacteriocins

Bacteriocins were purified as previously described using a multi-chromatographic procedure [60]. Briefly, 1 L supernatants from early stationary cultures of the recombinant lactococci were precipitated with (NH_4_)_2_SO_4_ (50%; *w*/*v*), desalted by gel filtration (PD-10 columns) and subjected to a cation-exchange (SP Sepharose Fast Flow), followed by a hydrophobic interaction (Octyl-Sepharose CL-4B) and reverse-phase chromatography in an ÄKTA purifier Reverse Phase Fast Protein Liquid Chromatography system (RP-FPLC), using the PepRPC HR 5/5 column. Fractions exhibiting the highest bacteriocin activity were pooled and re-chromatographed on the same column until chromatographically pure bacteriocin peptides were obtained. All chromatographic columns and equipment were obtained from GE Healthcare Life Sciences (Barcelona, Spain).

### 2.7. Mass Spectrometry (MS) and Multiple Reaction Monitoring (MRM) Analysis of Purified Peptide Fractions from Supernatants of the Recombinant L. lactis Strains

Purified RP-FPLC fractions from the supernatants of the recombinant lactococcal strains were subjected to matrix-assisted laser desorption–ionization time-of-flight mass spectrometry (MALDI-TOF MS) and multiple reaction monitoring liquid chromatography–electrospray ionization tandem mass spectrometry (MRM-LC-ESI-MS/MS) analyses at the Unidad de Proteómica (CAI Técnicas Biológicas, UCM). Briefly, 1 μL of eluted fractions were spotted onto a MALDI target plate and allowed to air-dry at room temperature. Then, 0.8 μL of a sinapic acid matrix (Sigma-Aldrich) in 30% acetonitrile and 0.3% trifluoroacetic acid was added and allowed to air-dry at room temperature. MALDI-TOF MS analyses were performed using a 4800 Plus Proteomics Analyzer MALDI-TOF/TOF mass spectrometer (Applied Biosystems/MDS Sciex, Toronto, Canada).

For the identification of bacteriocins, the MRM method evaluates a complex mixture of tryptic peptides that can be selectively detected by liquid chromatography coupled to electrospray MS. Briefly, the purified RP-FPLC fractions of interest were dried in Speed-vac and resuspended in 20 μL of 8 M urea. The samples were reduced by adding 10 mM of dithiothreitol for 45 min at 37 °C and alkylated with 55 mM of iodacetamide for 30 min in the dark. The urea was then diluted with 25 mM of ammonium bicarbonate to obtain a molarity of less than 2. When the pH was 8.5, digestion was performed by adding recombinant sequencing grade Trypsin (Roche Molecular Biochemicals, Branchburg, NJ, USA) 1:20 (*w*/*w*) and incubating at 37 °C. After 60 min, an aliquot was taken for the partial digestion of the sample. The rest was incubated overnight. The produced peptides were dried in Speed-vac and resuspended in 2% acetonitrile and 0, 1% formic acid. Skyline (64-bit), version 20.1, was used to build and optimize the MRM for the detection of the peptides of interest [61].

All analyses were performed on a LC-MS/MS Eksigent Nanoflow LC system coupled to a hybrid triple quadrupole/ion trap mass spectrometer, 5500 QTRAP (AB Sciex, Foster City, CA, USA), equipped with a nano electrospray interface operating in the positive ion mode. The MS/MS data were analyzed using Protein Pilot 4.5 software (AB Sciex) or MASCOT 2.3 (MatrixScience, London, UK) to identify the peptides against an in-house DataBase with the fasta sequences of the targeted proteins. The searches were performed assuming a digestion with trypsin with a maximum of 2 missed cleavages, a fragment ion mass tolerance of 0.6 Da and a parent ion tolerance of 0.15 Da. Peptide identifications based on the MS/MS data were accepted if they could be established at a CI of greater than 95% (*p* < 0.05). Data were then processed against the MRM-library on Skyline to ensure consistency between the transitions detected and the sequences of the peptides searched.

## 3. Results

### 3.1. Genetic Design and Cloning of Synthetic Genes That Drive the Heterologous Production of GarA and/or GarQ by Recombinant L. lactis Cells

In this work, synthetic genes containing the protein SP*_usp45_*, fused to mature GarA (*lgnA*) and its putative immunity protein GarAI (*lgnI*) (AI) encoded by *L. garvieae* 21881 [34], synthetic genes containing the protein SP*_usp45_* fused to mature GarQ (*garQ*) and its putative immunity protein GarQI (*lgnI*) (QI) encoded by *L. garvieae* BCC43578 [39], and synthetic genes containing the genetic fusions SP*_usp45_*::*lgnA*+*lgnI+garQ+garI* (AIQI) and SP*_usp45_*::*garQ*+*garI+lgnA+lgnI* (QIAI), were designed for cloning into the protein expression vectors pMG36c which carries the P*_32_*constitutive promoter, and in pNZ8048c with the inducible P*_nisA_* promoter. PCR-based amplifications of the synthesized gene fragments allowed for the generation of the PCR products shown in Table S1. Cloning the PCR products A, B, C and D in pMG36c resulted in the pMG36c-derived vectors pJFAI, pJFQI, pJFAIQI and pJFQIAI, and cloning the PCR product E in pNZ8048c resulted in the pNZ8048c-derived vector pNJFAI (Table 1). Similarly, the use of a novel, PCR-based, restriction-enzyme-free cloning method [59] for cloning fragments in plasmid pNZ8048 allowed for the construction of the pNZ8048c-derived vectors pNJFQI and pNJFQIAI (Table 1). In this study, no efforts were made to omit the presence of hypothetically redundant genes that encode putative immunity proteins in the designed synthetic gene fragments.

### 3.2. Antimicrobial Activity of the Recombinant L. lactis Strains as Determined by Their Direct Antagonistic Effect (SOAT) and the Antimicrobial Activity (ADT) of Their Cell-Free Supernatants

The transformation of recombinant plasmids into *L. lactis* subsp. *cremoris* NZ9000 showed that while the control NZ9000 (pMG36c) and NZ9000 (pNZ8048c) cells showed no antimicrobial activity against *L. garvieae* CF00021 or *P. damnosus* CECT4797, all the recombinant NZ9000-derived strains exhibited a measurable antagonistic effect against *L. garvieae* CF00021 (Table 2). Interestingly, results from both the SOAT and ADT tests showed that the recombinant strains NZ9000 (pJFAI) and NZ9000 (pNJFAI), which only encode the production of GarA, showed no antimicrobial activity against *P. damnosus* CECT4797.

**Figure 1:**
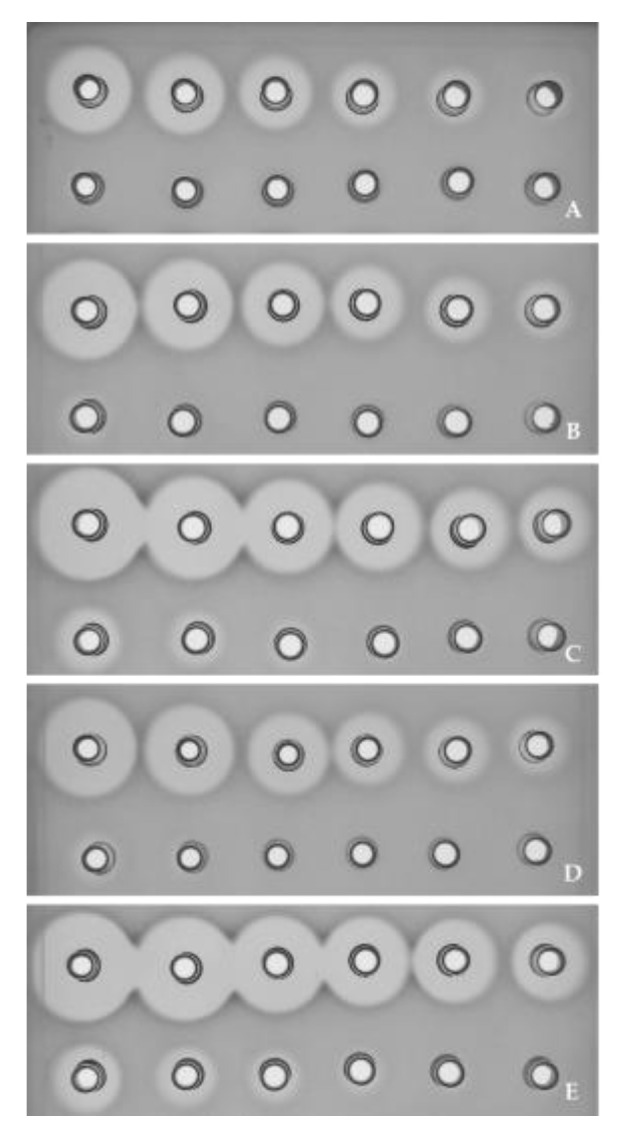
Antimicrobial activity of diluted supernatants of recombinant *L. lactis* strains as determined by an agar diffusion test (ADT) with *L. garvieae* CF00021 as the indicator microorganism. Supernatants from: *L. lactis* subsp. *cremoris* WA2-67 (pMG36c) (**A**), *L. lactis* subsp. *cremoris* WA2-67 (pJFAI) (**B**), *L. lactis* subsp. *cremoris* WA2-67 (pJFQI) (**C**), *L. lactis* subsp. *cremoris* WA2-67 (pJFAIQI) (**D**) and *L. lactis* subsp. *cremoris* WA2-67 (pJFQIAI) (**E**). Supernatants from recombinant strains in the upper lines were diluted 1, 2, 4, 8, 16 and 32 times, and in the bottom lines were diluted 64, 128, 256, 512, 1024 and 2048 times.

However, the recombinant strains derived from the native NisZ producer (*L. lactis* subsp*. cremoris* WA2-67) and the native NisA producers (*L. lactis* subsp. *lactis* DPC5598 and *L. lactis* subsp. *lactis* BB24) showed a higher antimicrobial activity against *P. damnosus* CECT4797 than *L. garvieae* CF00021. The strains that only encoded the production of GarA and/or GarQ but not NisZ or NisA (NZ9000 derivatives) showed a weaker antimicrobial activity against *P. damnosus* CECT4797. A closer evaluation of the antimicrobial activity of the NisZ-producing strain *L. lactis* subsp. *cremoris* WA2-67 suggested that the strains WA2-67 (pJFQI) and WA2-67 (pJFQIAI) displayed slightly larger halos of inhibition against both indicator strains than the rest of the recombinant strains evaluated (Table 2). Furthermore, the evaluation of the diluted supernatants of *L. lactis* subsp. *cremoris* WA2-67, which were transformed with pMG36c or the pMG36c-derived vectors, showed that the antimicrobial activity of the recombinant strains WA2-67 (pJFAI), WA2-67 (pJFQI), WA2-67 (pJFAIQI) and WA2-67 (pJFQIAI) was 2-, 8-, 2- and 16-fold higher, respectively, than the antimicrobial activity of *L. lactis* subsp. *cremoris* WA2-67 (pMG36c) (Figure 1).

The evaluation of the antimicrobial activity of the NisA-producing strain *L. lactis* subsp. *lactis* DPC5598 showed that recombinants encoding the production of either GarAI, GarQI, GarAI+GarQI or GarQI+GarAI displayed similar halos of inhibition to the control strains, DPC5598 (pMG36c) and DPC5598 (pNZ8048c), against both *P. damnosus* CECT4797 and *L. garvieae* CF00021. However, supernatants of the recombinant strains, derived from the NisA producer *L. lactis* subsp. *lactis* BB24, showed slightly larger inhibition halos against *L. garvieae* CF00021 than the control BB24 (pMG36c) and BB24 (pNZ8048) cells (Table 2).

### 3.3. Antimicrobial Activity of Recombinant L. lactis Strains against Different L. garvieae Strains

The antimicrobial activity of supernatants from all lactococcal strains was quantified against different virulent *L. garvieae* strains by using a more sensitive microtiter plate assay (MPA). Again, *L. lactis* subsp. *cremoris* NZ9000 (pMG36c) showed no antimicrobial activity against any of the *L. garvieae* strains evaluated, while the recombinant NZ9000-derived strains transformed with the constitutive pMG36c-derived vectors showed a weak antimicrobial activity against the virulent *L. garvieae* strains (Table 3). However, the NisZ-producing *L. lactis* subsp. *cremoris* WA2-67 (pMG36c) showed a much higher antimicrobial activity against *L. garvieae*. Remarkably, the antimicrobial activity determined for the recombinant WA2-67-derived strains transformed with the pMG36c-derived vectors to generate the WA2-67 (pJFAI), WA2-67 (pJFQI), WA2-67 (pJFAIQI) and WA2-67 (pJFQIAI) bacteriocin producers was 1.0- to 1.6-fold, 5.1- to 10.7-fold, 0.9- to 1.6-fold, and17.3- to 68.2-fold higher, respectively, than that of the control strain WA2-67 (pMG36c) (Table 3). The antimicrobial activity of the NisA producer *L. lactis* subsp. *lactis* DPC5598 recombinants was 0.5- to 1.2-fold higher than that of the control DPC5598 (pMG36c) strain against *L. garvieae*, while the antagonistic activity of the NisA producer *L. lactis* subsp. *lactis* BB24 recombinants was 1.3- to 3.7-fold higher than that of the control BB24 (pMG36c) strain against the same *L. garvieae* indicator strains (Table 3).

When the antimicrobial activity of the lactococcal strains that were transformed with the inducible pNZ8048c-derived vectors was determined, *L. lactis* subsp. *cremoris* NZ9000 (pNZ8048c) showed no antimicrobial activity against any of the *L. garvieae* strains, while the recombinant NZ9000 (pNJFAI), NZ9000 (pNJFQI), and NZ9000 (pNJQIAI) strains showed a 12.8- to 18.9-fold, 7.4- to 18.1-fold and 6.9- to 14.4-fold higher antimicrobial activity, respectively, than the cells transformed with the pMG36c-derived vectors (Table 4). However, the antimicrobial activity of the *L. lactis* subsp. *cremoris* WA2-67 (pNZ8048c) cells and their recombinant WA2-67 (pNJFAI), WA2-67 (pNJFQI) and WA2-67 (pNJFQIAI) strains showed a 0.7- to 0.9-fold, 0.5- to 0.8-fold, 0.1- to 0.2-fold and 0.01- to 0.08-fold lower antimicrobial activity, respectively, against the *L. garvieae* strains than the cells transformed with the pMG36c-derived vectors (Table 4). The antimicrobial activity of the NisA-producing *L. lactis* subsp. *lactis* DPC5598 transformed with the pNZ8048c-derived vectors was only slightly (0.9- to 2.0-fold) higher than the antimicrobial activity of the cells transformed with the pMG36c-derived vectors. Similarly, the antagonistic activity of the NisA producer *L. lactis* subsp. *lactis* BB24 recombinants transformed with the pNZ8048c-derived vectors was only slightly (0.6- to 1.9-fold) higher than cells transformed with the pMG36c-derived vectors (Table 4).

### 3.4. Purification of Bacteriocins Produced by L. lactis subsp. cremoris WA2-67 (pJFQI) and L. lactis subsp. cremoris WA2-67 (pJFQIAI)

The results regarding the purification to homogeneity of the bacteriocins in supernatants of the selected *L. lactis* subsp. *cremoris* recombinants are summarized in Table 5. The evaluation of the most active antimicrobial fractions after the first reversed-phase chromatography step (RP-FPLC) permitted the identification of two active fractions during the purification of the bacteriocins produced by *L. lactis* subsp. *cremoris* WA2-67 (pJFQI) and three active fractions during the purification of the bacteriocins produced by *L. lactis* subsp. *cremoris* WA2-67 (pJFQIAI). Although the antimicrobial activity of the eluted fractions was low, a significant increase in their specific antimicrobial activity was observed.

### 3.5. Mass Spectrometry (MS) and Multiple Reaction Monitoring (MRM) Analysis of the Purified Bacteriocin Fractions

MALDI-TOF MS analysis of fraction 8 and fraction 7, eluted during the RP-FPLC step of the purification of supernatants from *L. lactis* subsp. *cremoris* WA2-67 (pJFQI) and *L. lactis* subsp. *cremoris* WA2-67 (pJFQIAI), showed major peaks of 3331.4 Da and 3331.3 Da (Figure S1), respectively, matching the molecular mass described for NisZ. In addition, second peaks of 3349.3 Da and 3348.8 Da, respectively, likely correspond to the oxidation of the lanthionine ring of NisZ (Figure S1).

However, MALDI-TOF MS analysis of the eluted fraction 14 from the *L. lactis* subsp. *cremoris* WA2-67 (pJFQI), which encoded GarQ and NisZ, in addition to an analysis of fractions 9 and 12 from the *L. lactis* subsp. *cremoris* WA2-67 (pJFQIAI), which encoded GarA, GarQ and NisZ, could not identify the presence of the bacteriocins GarA and GarQ with predicted molecular masses of 4645.2 Da and 5340 Da, respectively, in the eluted fractions. Since this was a totally unexpected result, the fractions were subjected to MRM-LC-ESI-MS/MS analysis to determine the presence of the expected bacteriocins in the samples. In the MRM method, a series of target tryptic peptides and their associated transitions (fragments *m*/*z*) were predicted from the molecular masses and amino acid sequences of the bacteriocins GarA and GarQ. Each targeted peptide has a set of accompanying transitions which are then selectively detected in the second stage of the MS. A summary of the results obtained is shown in Table 6. MRM transitions were established and validated by tandem mass spectrometry (MS/MS). For each bacteriocin, two encrypted peptides were confidently detected in duplicate runs.

## 4. Discussion

Virulent *L. garvieae* are the etiological agents of a hyperacute hemorrhagic septicemia in fish, known as lactococcosis. They are also responsible for human pathologies due to their zoonotic character and potential presence in foods [31,33]. Bacteriophages and bacteriocins have potential as complementary strategies for combating *L. garvieae* in foods and fish [26,27], while bacteriocinogenic LAB could be evaluated for their potential use as probiotics, paraprobiotics and postbiotics in food, feed and other biotechnological applications [7,53,62]. The optimization of bacteriocin gene synthesis, expression and production helps the development of LAB as cell factories for the production and delivery of multiple bacteriocins [45,46,63]. The use of synthetic genes that match the codon usage of the producer organisms has a significant impact on gene expression levels and protein folding [47,64].

In this work, the transformation of *L. lactis* subsp. *cremoris* NZ9000 with pMG36c- or pNZ8048c-derived vectors demonstrated that the NZ9000 (pJFAI) and NZ9000 (pNJFAI) recombinant cells, which encode GarAI, showed antimicrobial activity against *L. garvieae* CF00021 but not against *P. damnosus* CECT4797 (Table 2), confirming their production of GarA and previous observations that this bacteriocin was only active against *L. garvieae* [34]. However, differences in the antimicrobial activity of recombinants derived from the NisZ producer *L. lactis* subsp. *cremoris* WA2-67, which was transformed with the pMG36c-derived vectors but not pNZ8048c-derived vectors, were observed against both indicator strains. The obtained results showed that the WA2-67 (pJFQI) cells exhibited larger halos of inhibition than the WA2-67 (pJFAI) cells, and that the WA2-67 (pJFQIAI) cells displayed the largest observed halos (Figure 1). These results suggest that the constitutive expression of GarQI is higher than GarAI or that the specific antimicrobial activity of GarQ is higher than that of GarA. Perhaps the transcription, processing and secretion from genes encoding GarQI+GarAI are more effective than from genes encoding GarAI+GarQI. On the other hand, no remarkable differences were found in the antimicrobial activity of the recombinants derived from the NisA producers transformed with the pMG36c-derived or the pNZ8048c-derived vectors, *L. lactis* subsp. *lactis* DPC5598 and *L. lactis* subsp. *lactis* BB24 (Table 2).

Due to the increasing interest that *L. garvieae* is attracting as not only a relevant bacterial pathogen but also as a zoonotic agent [29,65,66], the antimicrobial activity of the *L. lactis* recombinants was further evaluated and quantified against different virulent *L. garvieae* strains by using a more sensitive microplate assay (MPA). The obtained results showed that the *L. lactis* subsp. *cremoris* NZ9000 cells transformed with the pNZ8048c-derived vectors showed a 6.9- to 18.9-fold higher antimicrobial activity than the recombinant cells bearing the pMG36c-derived vectors (Table 3 and Table 4). The enhanced antimicrobial activity in cells with the nisin-inducible constructs may be due to copy number differences between pNZ8048c and pMG36c, but is more likely caused by the promoters used to drive gene expression [67,68]. Plasmid pNZ8048c contains the high-copy number heterogramic replicon of the lactococcal plasmid pSH71 with a unique *NcoI* cleavage site downstream of the *nisA* ribosome binding site (RBS), which is used for translational fusions inducible by NisA [23,69]. To optimize protein production, inducible systems are usually considered superior to constitutive expression systems since the former allow for the achievement of a sufficient biomass before the initiation of target protein expression [70]. The increased antimicrobial activity observed with the NisA-induced cells may also be ascribed to the short induction time for the production of GarA and/or GarQ (3 h), which most likely prevented the secreted bacteriocins from attaching to cell walls to form aggregates and/or to undergo protease degradations.

Moreover and quite remarkably, the antimicrobial activity of *L. lactis* subsp. *cremoris* WA2-67 (pMG36c) and the pMG36c-derived WA2-67 (pJFAI), WA2-67 (pJFQI), WA2-67 (pJFAIQI), and WA2-67 (pJFQIAI) strains showed a 1.0- to 1.6-fold, 5.1- to 10.7-fold, 0.9- to 1.6-fold and 17.3- to 68.2-fold higher antimicrobial activity, respectively, than the control WA2-67 (pMG36c) strain (Table 3). These results also indicate that the expression of QI increases the antimicrobial activity of the producer cells; however, the expression of AI has a much lower effect. Additionally, no increase in antimicrobial activity is observed when QI is expressed as the second of the two modules (AIQI). However, when QI is expressed as the first module (QIAI), a synergistic effect of both modules seems to occur regarding the very high antimicrobial activity of the producer cells. Thus, the AI in the AIQI module appears to prevent the QI from becoming active. However, when QI is expressed first in the QIAI module, the AI appears to synergistically increase the QI activity (Table 3). The pMG36c vector is a shuttle vector. It is based on the low-copy replication origin of pWV01 and is able to replicate in *Escherichia coli*, *Bacillus subtilis* and LAB, whereas the strong P*_32_* promoter drives the constitutive transcription of inserted genes into the multicloning site (MCS) of pUC18 [56]. From the results obtained, it may occur that, as previously suggested, the specific antimicrobial activity of GarA against *L. garvieae* is lower and/or its production and stability is less than that of GarQ. Additionally, besides the choice of vector and promoters, other factors such as the activation of quality control networks involving folding factors and housekeeping proteases, the oxidation of methionine to methionine-sulfoxide, bacteriocin self-aggregation and mRNA stability may affect bacteriocin production and activity from the recombinant hosts [46,71]. The coexpression of putative immunity genes may also increase the production of bacteriocins in heterologous hosts. These immunity proteins can act by either affecting bacteriocin pore formation or by perturbing the interaction between the bacteriocin and a membrane-located bacteriocin receptor, thereby preventing producer cells from being killed [16]. The expression in AIQI of LgnI before the expression of GarI could also affect producer protection against GarQ, thereby affecting growth and bacteriocin production by the producer cells. Importantly, *L. lactis* subsp. *cremoris* WA2-67 (pJFQI) and *L. lactis* subsp. *cremoris* WA2-67 (pJFQIAI) showed the highest antimicrobial activity against all virulent strains of *L. garvieae* evaluated (Table 3).

However, the antimicrobial activity of *L. lactis* subsp. *cremoris* WA2-67, which was transformed with the pNZ8048c-derived vectors, produced WA2-67-derivatives that showed a 0.01- to 0.9-fold lower antimicrobial activity, respectively, than the cells transformed with the pMG36c-derived vectors (Table 4). This was an unexpected result since, as previously described, inducible systems are often considered superior to constitutive expression systems for the optimization of protein production. Perhaps NisK and NisR, the two component signal transduction systems for the regulation of NisZ synthesis in *L. lactis* subsp. *cremoris* WA2-67, do not fully activate transcription of the P*_nisA_* present in pNZ8048c. It could be also possible that levels of phosphorylated NisR may be not enough to drive the activation of two independent P*_nis_* promoters which, in addition, derive from two different *Lactoccocus lactis* subspecies: *cremoris* and *lactis*, respectively. Alternatively, NisZ may not be as efficient as NisA for an interaction with the NisK produced by *L. lactis* subsp. *cremoris* WA2-67, thus constraining the induction of transcription of P*_nisA_* in pNZ8048c by blocking NisZ with NisI and NisEFG.

The antimicrobial activity of the NisA-producers *L. lactis* subsp. *lactis* DPC5598 and *L. lactis* subsp. *lactis* BB24 was slightly higher for the cells transformed with the pNZ8048c- than the pMG36c-derived vectors, suggesting that NisA is a better inducer than NisZ for the activation of the transcription of P*_nisA_* in pNZ8048c (Table 3 and Table 4). However, the antimicrobial activity of the DPC5598-derived recombinants under constitutive or inducible conditions was lower during multi-bacteriocin production. This was probably due to the high energy and metabolic cost linked to plasmid maintenance and replication, to the secretion stress associated with bacteriocin overproduction and/or to the synthesis of proteinases for the elimination of misfolded proteins [72,73,74]. Differences in the antimicrobial activity of these strains and those from the *L. lactis* subsp. *cremoris* WA2-67 transformed with the pMG36c-derived vectors may be also ascribed to yet-unknown genetic and/or metabolic differences between the strains. *L. lactis* subsp. *lactis* DPC5598 was selected as a potential multi-bacteriocin-producing host because it is a plasmid-free derivative of an industrial strain that is extensively used in fermented dairy products due to its phage insensitivity and fast acid-producing ability [54]. *L. lactis* subsp. *lactis* BB24 is a fermented, meat-derived isolate widely used as an efficient host for the heterologous production of bacteriocins [45,55]. Both multi-bacteriocin producers should be considered for their potential evaluation as probiotics, paraprobiotics and/or postbiotics reducing the increasing presence of virulent and zoonotic *L. garvieae* in selected milk and meat substrates, respectively [31,75].

The MALDI-TOF MS analysis of purified eluted fractions from supernatants of the most active antimicrobial strains, *L. lactis* subsp. *cremoris* WA2-67 (pJFQI) and *L. lactis* subsp. *cremoris* WA2-67 (pJFQIAI) (Table 5), allowed for the detection of NisZ in supernatants of the producer strains (Figure S1), suggesting that this bacteriocin is appropriately processed and transported out of the producer cells. However, the presence of GarA and GarQ could not be detected, suggesting interactions of the bacteriocins with unknown biological compounds, or a low amount and recovery of the bacteriocins during their purification to homogeneity [47,64]. However, bacteriocins in the purified fractions were suitable for MRM evaluation and data analysis, which is an emerging targeted proteomics workflow and a highly selective and sensitive method for detecting peptides in the low ng/mL to sub-ng/mL range concentrations [64,76,77]. When the purified fractions were subjected to MRM-LC-ESI-MS/MS analysis, two encrypted peptides were confidently (99%) detected. The detected peptides were confirmed by MS/MS, and at least four transitions were identified for each (Table 6). The peptide fragments covered 44% of the sequence of GarQ, while the coverage was 21% for GarA. These relatively low-coverage percentages are related to the reduced presence of lysine and arginine (trypsin cleavage sites) in GarA and GarQ, which significatively reduces the number of potentially identifiable target peptides.

Previous studies by our research group identified probiotic features of the native or wild-type NisZ producer *L. lactis* subsp. *cremoris* WA2-67 such as a potent antimicrobial activity against ichthyopathogens, survival in fresh water and the gastrointestinal tract of trout, a resistance to bile and low pH, and an improved colonization ability with respect to the intestinal trout mucosa [26,53,78]. Further in silico analyses of the whole-genome sequence (WGS) of this strain also identified other potential probiotic traits such as the production of vitamins and amino acids, adhesion/aggregation and stress resistance factors and the absence of transferable antibiotic resistance determinants and genes encoding detrimental enzymatic activities or potential virulence factors [79]. Other studies performed by our group demonstrated the effectiveness of *L. lactis* subsp. *cremoris* WA2-67 to protect rainbow trout in vivo against infection of the virulent *L. garvieae* and the relevance of NisZ production as an anti-infective mechanism [53].

The work described in this study constitutes the first report on the design of multi-bacteriocinogenic *L. lactis* subsp. *cremoris* WA2-67 strains with a high antimicrobial activity against virulent *L. garvieae* and a promising role as probiotics, paraprobiotics and/or postbiotics in food, feed and other biotechnological applications. The evaluation of bioengineered strains as probiotics is subjected to approval by regulatory authorities and is performed under strict biological conditions. However, the number of reports on the evaluation of bioengineered bacterial strains as probiotics (live cells), paraprobiotics (dead, non-viable cells) and postbiotics (physical-, chemical- or enzymatic-lysis of probiotic cells) is increasing [7,80,81]. Accordingly, experiments are being planned to evaluate the in vitro effect of *L. lactis* subsp. *cremoris* WA2-67 (pJFQI) and *L. lactis* subsp. *cremoris* WA2-67 (pJFQIAI) on rainbow trout intestinal epithelial cells (RTgutGC) for a transcriptional analysis of several immune, intestinal, barrier-integrity and homeostasis genes and the induction of antimicrobial peptides (AMPs), as well as for their effect on the in vivo modulation of the intestinal microbiota and immune response of rainbow trout (*Oncorhynchus mykiss*, Walbaum) and turbot (*Scophthalmus maximus*).

## 5. Conclusions

The design of synthetic genes and their cloning into protein expression vectors bearing constitutive or inducible promoters has allowed for the production and functional expression of GarA and/or GarQ by *L. lactis* subsp*. lactis* and *L. lactis* subsp. *cremoris* strains. Most importantly, *L. lactis* subsp*. cremoris* WA2-67, transformed with the pMG36c-derived vectors, allowed for the obtention of *L. lactis* subsp*. cremoris* WA2-67 (pJFQI), a producer of GarQ and NisZ, and *L. lactis* subsp*. cremoris* WA2-67 (pJFQIAI), a producer of GarA, GarQ and NisZ, with a much higher antimicrobial activity (5.1- to 10.7-fold and 17.3- to 68.2-fold, respectively) against virulent *L. garvieae* than the rest of the *L. lactis* strains evaluated. The concerted use of a sensitive microtiter plate assay (MPA) for the quantification of the antimicrobial activity of supernatants, the use of a multi-chromatographic procedure for the purification of bacteriocins to homogeneity, and the use of a MALDI-TOF MS multiple reaction monitoring (MRM-LC-ESI-MS/MS) analysis of the purified bacteriocins are unavoidable and possibly irreplaceable tools for the identification and characterization of the bacteriocins produced by *L. lactis* subsp*. cremoris* WA2-67 (pJFQI) and *L. lactis* subsp*. cremoris* WA2-67 (pJFQIAI).

## Figures and Tables

**Table 1:** Bacterial strains and plasmids used in this study.

Strain or Plasmid	Description ^a^	Source and/or Reference ^b^
Strains		
*Lactococcus lactis* subsp. *cremoris* NZ9000	Host strain; non-bacteriocin producer; pepN::*nis*RK.	NIZO, [52]
*Lactococcus lactis* subsp. *cremoris* WA2-67	Host strain; fish origin; nisin Z producer.	SD- NUTRYCIAL, [53]
*Lactococcus lactis* subsp. *lactis* DPC5598	Host strain; milk origin; nisin A producer. Plasmid-free derivative of DPC4268.	DPC, [54]
*Lactococcus lactis* subsp. *lactis* BB24	Host strain; meat origin; nisin A producer.	SD- NUTRYCIAL, [55]
*Lactococcus garvieae* CF00021	SOAT, ADT, MPA indicator	CEFAS-BCC
*Lactococcus garvieae* CF01144	MPA indicator	CEFAS-BCC
*Lactococcus garvieae* CLG4	MPA indicator	LFP-UNIZAR
*Lactococcus garvieae* CLG5	MPA indicator	LFP-UNIZAR
*Lactococcus garvieae* CLFP28/06	MPA indicator	LFP-UNIZAR
*Lactococcus garvieae* JIP29/99	MPA indicator	Jouy-en-Josas SC
*Pediococcus damnosus* CECT4797	SOAT, ADT indicator	CECT
Plasmids		
pMG36c	Cm^r^; pMG36e derivative; constitutive expression vector carrying the P*_32_* promoter.	RUG-MG, [56]
pNZ8048c	Cm^r^; inducible expression vector carrying the *nisA* promoter.	NIZO, [52]
pJFAI	Cm^r^, pMG36c derivative carrying the PCR product A (P*_32_* ribosome binding site and the SP*_usp45_* fused to mature *lgnA* and *lgnI*).	This work
pJFQI	Cm^r^, pMG36c derivative carrying the PCR product B (P*_32_* ribosome binding site and the SP*_usp45_* fused to mature *garQ* and *garI*).	This work
pJFAIQI	Cm^r^, pMG36c derivative carrying the PCR product C (P*_32_* ribosome binding site and the SP*_usp45_* fused to mature *lgnA* and *lgnI* and mature *garQ* and *garI*).	This work
pJFQIAI	Cm^r^, pMG36c derivative carrying the PCR product D (P*_32_* ribosome binding site and the SP*_usp45_* fused to mature *garQ* and *garI* and mature *lgnA* and *lgnI*).	This work
pNJFAI	Cm^r^, pNZ8048c derivative carrying the PCR product E (SP*_usp45_* fused to mature *lgnA* and *lgnI*).	This work
pNJFQI	Cm^r^, pNZ8048c derivative corresponding to the PCR product J (SP*_usp45_* fused to mature *garQ* and *garI*).	This work
pNJFQIAI	Cm^r^, pNZ8048c derivative corresponding to the PCR product K (SP*_usp45_* fused to mature *garQ* and *garI* and mature *lgnA* and *lgnI*).	This work

^a^ SOAT— stab-on-agar test; ADT—agar diffusion test; MPA—microtiter plate assay; Cm^r^—chloramphenicol resistance. ^b^ NIZO—Department of Biophysical Chemistry, NIZO Food Research (Ede, The Netherlands); SD-NUTRYCIAL—Sección Departamental de Nutrición y Ciencia de los Alimentos, Facultad de Veterinaria, Universidad Complutense de Madrid (Madrid, Spain); DPC—Teagasc Dairy Products Research Centre, Moorepark, Fermoy, Co., (Cork, Ireland); CEFAS-BCC— Centre for Environment Fisheries and Aquaculture Science-Bacterial Culture Collection (Suffolk, United Kingdom); LFP-UNIZAR—Laboratory of Fish Pathology, Universidad de Zaragoza (Zaragoza, Spain); Jouy-en-Josas SC—Jouy-en-Josas Strain Collection, INRAE (Jouy-en-Josas, France); CECT—Spanish Type Culture Collection, Universidad de Valencia (Valencia, Spain); RUG-MG—Department of Molecular Genetics, University of Groningen (Haren, The Netherlands).

**Table 2:** Direct antimicrobial activity and extracellular antimicrobial activity of recombinant *L. lactis* strains, producing garvicin A (GarA) and/or garvicin Q (GarQ), against different bacterial indicators.

Strain	SOAT ^a^		ADT ^b^	
	*L. garvieae* CF00021	*P. damnosus* CECT4797	*L. garvieae* CF00021	*P. damnosus* CECT4797
*L. lactis* subsp. *cremoris*				
**NZ9000 (pMG36c)**	**(-)**	**(-)**	**(-)**	**(-)**
NZ9000 (pJFAI)	6.7	(-)	10.1	(-)
NZ9000 (pJFQI)	6.4	6.0	10.3	8.6
NZ9000 (pJFAIQI)	6.6	5.8	10.4	8.9
NZ9000 (pJFQIAI)	6.8	5.9	10.2	8.8
**NZ9000 (pNZ8048c)**	**(-)**	**(-)**	**(-)**	**(-)**
NZ9000 (pNJFAI)	8.6	(-)	11.9	(-)
NZ9000 (pNJFQI)	8.3	7.1	12.2	11.2
NZ9000 (pNJFQIAI)	8.1	6.5	11.7	9.5
**WA2-67 (pMG36c)**	**12.6**	**20.5**	**13.7**	**21.7**
WA2-67 (pJFAI)	12.8	20.4	14.0	21.8
WA2-67 (pJFQI)	13.6	21.3	15.6	23.2
WA2-67 (pJFAIQI)	12.7	20.1	13.8	21.7
WA2-67 (pJFQIAI)	14.8	23.0	16.9	25.4
**WA2-67 (pNZ8048c)**	**12.3**	**20.5**	**13.6**	**21.3**
WA2-67 (pNJFAI)	12.7	20.1	13.8	21.7
WA2-67 (pNJFQI)	12.2	20.8	14.0	22.2
WA2-67 (pNJFQIAI)	12.3	20.3	13.8	22.0
*L. lactis* subsp. *lactis*				
**DPC5598 (pMG36c)**	**7.2**	**9.0**	**13.7**	**20.0**
DPC5598 (pJFAI)	7.6	8.7	13.5	19.4
DPC5598 (pJFQI)	7.5	8.9	13.6	19.7
DPC5598 (pJFAIQI)	6.8	7.6	12.8	18.0
DPC5598 (pJFQIAI)	6.2	6.5	11.9	16.7
**DPC5598 (pNZ8048c)**	**7.6**	**9.5**	**14.0**	**20.6**
DPC5598 (pNJFAI)	7.8	9.1	14.5	21.3
DPC5598 (pNJFQI)	7.3	9.4	14.8	22.0
DPC5598 (pNJFQIAI)	6.8	8.6	12.5	19.1
**BB24 (pMG36c)**	**7.9**	**9.7**	**12.4**	**18.6**
BB24 (pJFAI)	9.9	10.0	14.5	18.3
BB24 (pJFQI)	9.4	13.4	14.6	20.5
BB24 (pJFAIQI)	9.7	13.8	14.2	19.9
BB24 (pJFQIAI)	9.6	13.9	14.3	20.2
**BB24 (pNZ8048c)**	**8.5**	**10.1**	**13.0**	**18.7**
BB24 (pNJFAI)	10.3	13.7	15.0	19.3
BB24 (pNJFQI)	10.7	14.2	15.1	20.4
BB24 (pNJFQIAI)	10.2	14.6	15.0	20.0

^a^ Direct antimicrobial activity as determined by a stab-on-agar test (SOAT) and ^b^ extracellular antimicrobial activity as determined by an agar diffusion test (ADT). Both are expressed as halos of growth inhibition (diameter in mm). Most of the data are means from two independent determinations in triplicate. Control strains are in bold. (-)—no activity.

**Table 3:** Antimicrobial activity ^a^ of supernatants from recombinant *L. lactis* strains transformed with pMG36c-derived plasmids against virulent *L. garvieae* strains.

Strain	*L. garvieae*					
	CF00021	CF01144	CLG4	CLG5	CLFP28/06	JIP29/99
*L. lactis* subsp. *cremoris*						
**NZ9000 (pMG36c)**	**(-)**	**(-)**	**(-)**	**(-)**	**(-)**	**(-)**
NZ9000 (pJFAI)	88	90	76	69	88	85
NZ9000 (pJFQI)	98	143	112	106	97	100
NZ9000 (pJFAIQI)	121	169	151	153	149	140
NZ9000 (pJFQIAI)	126	145	101	167	107	108
**WA2-67 (pMG36c)**	**2199**	**1035**	**1148**	**391**	**1087**	**521**
WA2-67 (pJFAI)	2276	1469	1920	475	1566	744
WA2-67 (pJFQI)	11,798	11,082	5935	4201	8864	4821
WA2-67 (pJFAIQI)	2193	1749	1825	468	1637	847
WA2-67 (pJFQIAI)	150,033	19,571	28,362	6780	33,114	9412
*L. lactis* subsp. *lactis*						
**DPC5598 (pMG36c)**	**1808**	**1271**	**1865**	**139**	**1042**	**855**
DPC5598 (pJFAI)	1529	1326	2133	198	1318	960
DPC5598 (pJFQI)	1770	1338	1976	129	1288	1066
DPC5598 (pJFAIQI)	1096	1068	1312	92	1064	905
DPC5598 (pJFQIAI)	1033	659	1489	81	1055	815
**BB24 (pMG36c)**	**1123**	**790**	**443**	**115**	**1479**	**514**
BB24 (pJFAI)	2385	2480	1617	203	2036	1105
BB24 (pJFQI)	2532	2530	1725	202	3115	1243
BB24 (pJFAIQI)	2238	2756	1786	204	2615	1459
BB24 (pJFQIAI)	2535	2699	1641	196	3166	1317

^a^ Antimicrobial activity as determined by a microtiter plate assay (MPA) and expressed in bacteriocin units per milligram cell dry weight (BU/mg cdw). Most of the data are means from two independent determinations in triplicate. Control strains are in bold. (-)—no activity.

**Table 4:** Antimicrobial activity ^a^ of supernatants from recombinant *L. lactis* strains transformed with pNZ8048c-derived plasmids against virulent *L. garvieae* strains.

Strain	*L. garvieae*					
	CF00021	CF01144	CLG4	CLG5	CLFP28/06	JIP29/99
*L. lactis* subsp. *cremoris*						
**NZ9000 (pNZ8048c)**	**(-)**	**(-)**	**(-)**	**(-)**	**(-)**	**(-)**
NZ9000 (pNJFAI)	1126	997	1440	1295	1600	1263
NZ9000 (pNJFQI)	1214	1069	1395	1194	1440	1220
NZ9000 (pNJFQIAI)	1051	1188	1297	1167	1541	1255
**WA2-67 (pNZ8048c)**	**1668**	**1002**	**897**	**364**	**987**	**369**
WA2-67 (pNJFAI)	1853	989	1000	412	902	385
WA2-67 (pNJFQI)	2150	1351	1237	565	1163	486
WA2-67 (pNJFQIAI)	2021	1223	1149	595	1245	491
*L. lactis* subsp. *lactis*						
**DPC5598 (pNZ8048c)**	**2123**	**1258**	**2317**	**198**	**1097**	**1096**
DPC5598 (pNJFAI)	2760	2699	3443	317	1610	1770
DPC5598 (pNJFQI)	2605	1496	2334	266	1499	1492
DPC5598 (pNJFQIAI)	1150	880	1800	98	1480	920
**BB24 (pNZ8048c)**	**1203**	**693**	**397**	**158**	**1022**	**685**
BB24 (pNJFAI)	3398	3102	2340	339	2989	1597
BB24 (pNJFQI)	3705	3663	2732	397	4172	1603
BB24 (pNJFQIAI)	3502	3375	2541	299	5016	2001

^a^ Antimicrobial activity as determined by a microtiter plate assay (MPA) and expressed in bacteriocin units per milligram cell dry weight (BU/mg cdw). Most of the data are means from two independent determinations in triplicate. Control strains are in bold. (-)—no activity.

**Table 5:** Purification of NisZ and GarQ produced by *L. lactis* subsp. *cremoris* WA2-67 (pJFQI) and purification of NisZ, GarA and GarQ produced by *L. lactis* subsp. *cremoris* WA2-67 (pJFQIAI).

Purification Stage	Volume (mL)	Total A_254_ ^a^	Total Antimicrobial Activity (BU) ^b^	Specific Antimicrobial Activity (BU/A_254_) ^c^	Increase in Specific Antimicrobial Activity (Fold) ^d^	Recovery Antimicrobial Activity (%)
***L. lactis*** **subsp*. cremoris*** **WA2-67 (pJFQI)**						
Culture supernatant	1000	30,500	10.6 × 10^6^	347	1	100
Ammonium sulfate precipitation	100	1930	12.3 × 10^6^	6370	18	116
Gel filtration chromatography	185	872	2.1 × 10^6^	2410	7	20
Cation-exchange chromatography	50	75	11.3 × 10^6^	150,670	434	107
Hydrophobic-interaction chromatography	15	8.4	1.0 × 10^6^	119,050	343	9
Reversed-phase chromatography						
Fraction 8	0.300	0.325	50.0 × 10^3^	153,850	443	0.5
Fraction 14	0.400	0.056	2.3 × 10^3^	41,070	118	0.02
***L. lactis*** **subsp. *cremoris* WA2-67 (pJFQIAI)**						
Culture supernatant	1000	28,400	1.4 × 10^8^	4930	1	100
Ammonium sulfate precipitation	100	1510	1.5 × 10^9^	993,380	201	1071
Gel filtration chromatography	185	832	2.3 × 10^8^	276,440	56	164
Cation-exchange chromatography	50	119	1.8 × 10^9^	15,130	3	1286
Hydrophobic-interaction chromatography	15	13.8	16.1 × 10^6^	1,166,670	237	11.5
Reversed-phase chromatography						
Fraction 7	0.300	0.125	21,400	171,200	35	0.015
Fraction 9	0.450	0.218	2200	10,100	2	0.0016
Fraction 12	0.500	0.040	200	5000	1	0.00014

^a^ Absorbance at 254 nm (A_254_) multiplied by the volume in milliliters. ^b^ Antimicrobial activity in bacteriocin units per milliliter (BU/mL), determined by an MPA against *L. garvieae* CF00021 and multiplied by the total volume in milliliters. ^c^ Specific antimicrobial activity expressed as the total antimicrobial activity (BU) divided by the total A_254_. ^d^ Specific antimicrobial activity of a fraction (BU/A_254_) divided by the specific antimicrobial activity of the initial culture supernatant (BU/A_254_).

**Table 6:** Peptide fragments identified by MRM-LC-ESI-MS/MS (QTRAP) in eluted purified fractions from supernatants of *L. lactis* subsp. *cremoris* WA2-67 (pJFQI) and *L. lactis* subsp. *cremoris* WA2-67 (pJFQIAI).

Strain	Fraction	Peptide Sequence	Precursor MW	Detected *m*/*z*	Retention Time	Detected Transitions	MS/MS Confirmation
***L. lactis* subsp. *cremoris***							
**WA2-67 (pJFQI)**	14	**GarQ**					
		EYHLMNGANGYLTR	1638.76	546.92 (+3)	20.1	4	+
		VNGKYVYR	998.54	499.77 (+2)	8.7	4	+
**WA2-67 (pJFQIAI)**	9	**GarQ**					
		EYHLMNGANGYLTR	1638.76	546.92 (+3)	21.3	4	+
		VNGKYVYR	998.54	499.77 (+2)	10.4	4	+
	12	**GarA**					
		GKINQYRPY	1138.60	569.8 (+3)	14.7	4	+
		INQYRPY	953.48	477.24 (+2)	15.6	5	+

Amino acid sequence of GarQ: EYHLMNGANGYLT**R**VNG**K**YVY**R**VT**K**DPVSAVFGVISNGWGSAGAGFGPQH and GarA: IGGALGNALNGLGTWANMMNGGGFVNQWQVYAN**K**G**K**INQY**R**. PY. Lysine (K) and arginine (R) residues for hydrolysis by trypsin, in bold. +, positive confirmation.

## Data Availability

Data is contained within the article or supplementary material.

## Supplementary Materials

The following supporting information can be downloaded at: https://www.mdpi.com/article/10.3390/foods12051063/s1, Table S1: Primers and PCR products used in this study; Figure S1: MALDI-TOF MS analysis of purified NisZ from *Lactococcus lactis* subsp. *cremoris* WA2-67 (pJFQI) (A) and *Lactococcus lactis* subsp. *cremoris* WA2-67 (pJFQIAI (B). Numbers indicate the molecular mass in Daltons of major peptide fragments.
