# Supplementary material for: Design of Lactococcus lactis Strains Producing Garvicin A and/or Garvicin Q, Either Alone or Together with Nisin A or Nisin Z and High Antimicrobial Activity against Lactococcus garvieae
Source: Foods. 2023 Mar 2;12(5):1063. doi: 10.3390/foods12051063 (PMC10000435; doi:10.3390/foods12051063)
Supplement: Supplementary file 1 [file foods-12-01063-s001.zip › foods-2170420-supplementary.pdf]

Table S1. Primers and PCR products used in this study.

Primer or PCR product	Nucleotide sequence (5′–3′) or description[a]	PCR product amplified
**Primer**		
GAR-F	CATA*GAGCTC*TGTAA**GGAGG**	A, B, C, D
GARAIM-R	AAGTT*AAGCTT*GTATTACATAG	A, D, E
GARQIM-R	GTT*AAGCTT*GTATCAGTCTAAG	B, C
MGPJ-F	TGGATTTTTGTGAGCTTGGA	
MGPJ-R	TTTGTCGCTAGTACCGGTTG	
GARF-BSPHI	ATCCACTCA*TCATGA*AAAAAAAGATTATCTC	E
NZA1-F	CCCTGACGAAAGTCGACGGCAATAGTTACC	F, J, K
NZA2-R	CATGGTGAGTGCCTCCTTATAATTTATTTTG	F
NZC1-F	CTTTCTTTGAACCAAAATTAGAAAACCAAG	G
NZC2-R	AATAAGGGTAACTATTGCCGTCGACTTTCG	G, J, K
QB1-F	CAAAATAAATTATAAGGAGGCACTCACCATGAAAAAAAAGATTATCTCAG	H
QB2-R	CTTGGTTTTCTAATTTTGGTTCAAAGAAAGCTTGTATCAGTCTAAGTAAC	H
QAB1-F	CAAAATAAATTATAAGGAGGCACTCACCATGAAAAAAAAAAATCATTTCAG	I
QAB2-R	CTTGGTTTTCTAATTTTGGTTCAAAGAAAGCTTGTATTACATTGAAAATG	I
NZPJ-F	TGTCGATAACGCGAGCATAA	
NZPJ-R	CAAAGCAACACGTGCTGTAA	
**PCR product**		
A	544-bp *Sac*I/*Hind*III fragment containing the P_{32} ribosome binding site and the SP_{usp45} fused to mature *lgnA* and *lgnI*	
B	617-bp *Sac*I/*Hind*III fragment containing the P_{32} ribosome binding site and the SP_{usp45} fused to mature *garQ* and *garI*	
C	1,139-bp *Sac*I/*Hind*III fragment containing the P_{32} ribosome binding site and the SP_{usp45} fused to mature *lgnA* and *lgnI*, and mature *garQ* and *garI*	
D	1,139-bp *Sac*I/*Hind*III fragment containing the P_{32} ribosome binding site and the SP_{usp45} fused to mature *garQ* and *garI*, and mature *lgnA* and *lgnI*	
E	524-bp *Bsp*HI/*Hind*III fragment containing the SP_{usp45} fused to mature *lgnA* and *lgnI*	
F	1,254-bp fragment of pNZ8048c (overlaps PCR product G)	
G	2,089-bp fragment of pNZ8048c (overlaps PCR product F)	
H	636-bp fragment containing the SP_{usp45} fused to mature *garQ* and *garI* (overlaps PCR products F and G)	
I	1,158-bp fragment containing the SP_{usp45} fused to mature *garQ* and *garI*, and mature *lgnA* and *lgnI* (overlaps PCR products F and G)	
J	3,888-bp circular fragment (corresponding to plasmid pNJFQI)	
K	4,410-bp circular fragment (corresponding to plasmid pNJFQIAI)	

[a] Cleavage sites for restriction enzymes are italicized; P_{32} ribosome binding site is shown in bold.

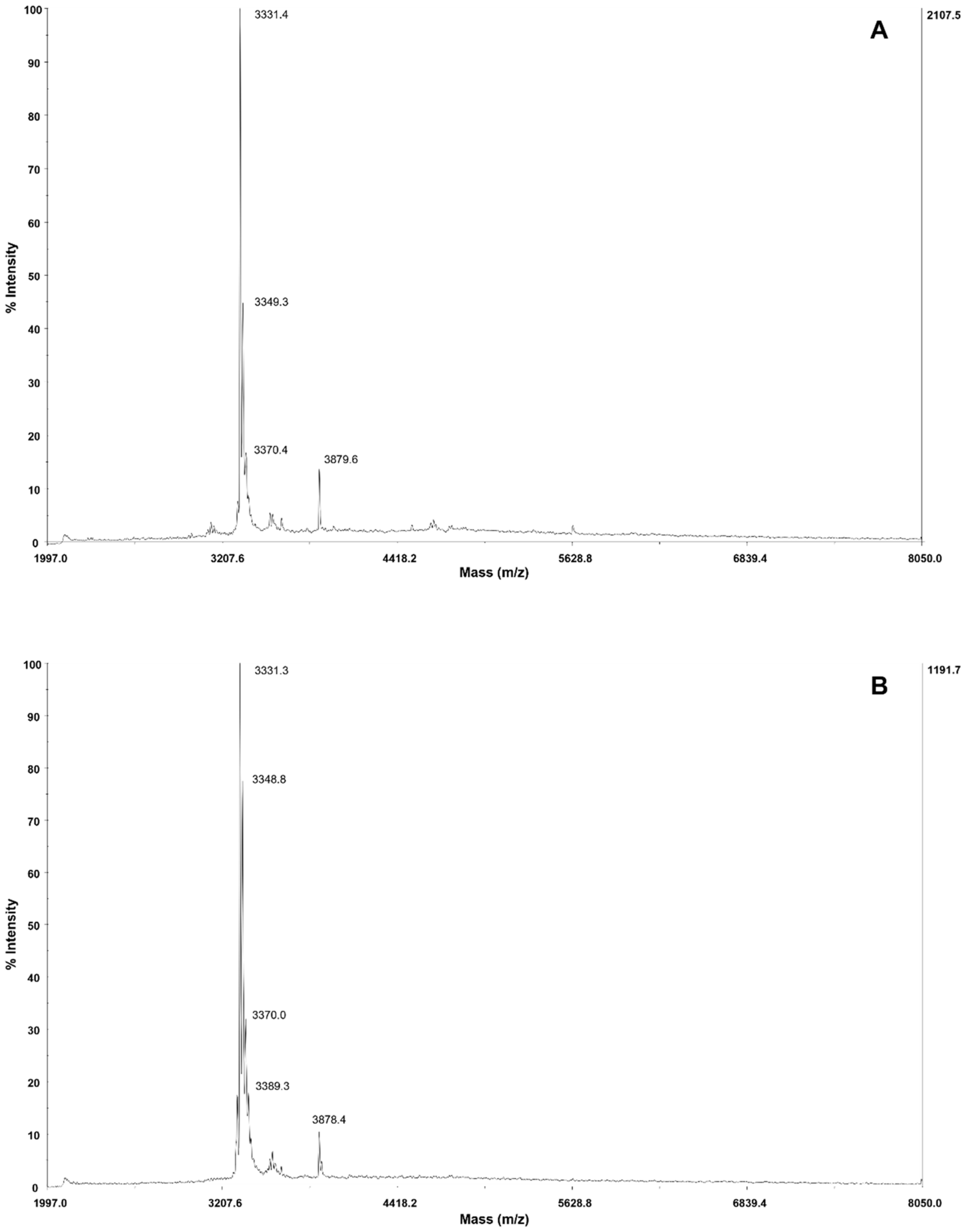

Figure S1. MALDI-TOF MS analysis of purified NisZ from *Lactococcus lactis* subsp. *cremoris* WA2-67 (pJFQI) (A), and *Lactococcus lactis* subsp. *cremoris* WA2-67 (pJFQIAI) (B). Numbers indicate the molecular mass in daltons of major peptide fragments.
